# The Spectrum of Pediatric Cardiac Procedures and Their Outcomes: A Six-month Report from the Largest Cardiac Facility in Sindh, Pakistan

**DOI:** 10.7759/cureus.5339

**Published:** 2019-08-07

**Authors:** Rajab A Khokhar, Murtaza A Gowa, Sohail K Bangash, Amber Tahir

**Affiliations:** 1 Pediatric Critical Care, National Institute of Cardiovascular Diseases, Karachi, PAK; 2 Pediatric Critical Care, National Institute of Child Health, Karachi, PAK; 3 Pediatric Cardiac Surgery, National Institute of Cardiovascular Diseases, Karachi, PAK; 4 Internal Medicine, Dow University of Health Sciences, Karachi, PAK

**Keywords:** pediatric cardiac critical care, tetralogy of fallot, pediatric cardiac surgery, national institute of cardiovascular diseases nicvd, congenital heart disease, pediatric intensive care unit, retrospective analysis, septal defects, ventricular septal defect, pakistan

## Abstract

Introduction

Congenital heart disease (CHD) is the most common birth defect globally, with low-to-middle income Asian countries registering the highest incidence. Every year, 60,000 babies are born with varying severity of CHD in Pakistan. But the country has only three pediatric intensive care units (PICU) fully dedicated to child cardiac surgery patients. The focus of this study is to analyze the spectrum of pediatric cardiac surgical procedures performed for the management of CHD and their outcomes in a cardiac PICU in Pakistan.

Methods

In this analysis, all surgical records of children admitted to the PICU of National Institute of Cardiovascular Diseases (NICVD), Karachi, from October 2018 to March 2019 were included. It is a 14-bed, state-of-the-art cardiac PICU, which provides high-quality care to critical post-surgical patients.

Results

The surgical records of 537 patients were extracted for the purpose of our study, which accounted for 89.5 of post-operative patients admitted in the PICU per month and three per day. Tetralogy of Fallot (TOF) was the most commonly treated anomaly (n = 161; 29.9%) in the facility, followed by ventricular septal defect (n = 107; 19.9%). The overall mortality rate was 5.4% (n = 29), out of which 27.5% (n = 8) were TOF-related.

Conclusions

There is a very high burden of patients on the cardiac PICUs in low-to-middle income Asian countries. Despite the lack of resources, the high- quality care provided by pediatric cardiac critical-care specialists at these PICUs has ensured favorable outcomes and a mortality rate as low as that in any of the developed countries.

## Introduction

Globally, congenital heart disease (CHD) is the most common birth defect. CHD is defined as structural anomalies of the walls of the heart, chamber septation, the heart valves, and/or great vessels, which are present at the time of birth. It may or may not be detected at the time of birth [1].

The literature has reported various statistics regarding the global incidence of CHD. In a recent meta-analysis of 260 studies, the mean prevalence of CHD from 2010-17 was reported to be 9.4 per 1,000 live births [2]. When the prevalence of CHD in different regions was compared, Asia reported the highest prevalence: 9.3 per 1,000 live births; with more pulmonary outflow obstructions and less left ventricular outflow tract obstructions as compared to other parts of the world [3].

There is a rising trend of CHDs in developing and low-to-middle income countries due to worsening maternal health. The modifiable risk factors reported for developing countries include teenage pregnancy, consanguineous marriage, overt diabetes mellitus, maternal obesity, maternal stress, history of previous CHDs/other congenital anomalies, non-intake of folic acid in the first trimester, and advanced maternal as well as paternal age [4,5]. There are no population-based statistics of CHD prevalence from countries like Pakistan, and the latest incidence reported dates back to 1997 (four per 1,000 live births) [6]. The major reason for the lack of recent literature is the prevalence of home deliveries and non-availability of experienced pediatricians in primary and secondary care maternity setups for early detection. Neonatal screening is not common. Apart from contributing to the absence of a real picture, these factors have also been the major reasons behind delayed presentation and diagnosis and, ultimately, worsening outcomes [1].

According to a report published in 2017, 60,000 babies are born with varying severity of CHD in Pakistan every year. The country has only four facilities that can surgically manage these children. Consequently, around 60% of Pakistani children with CHD are unable to live beyond the first few years of their lives [7]. The relatively higher mortality rate among children with CHD in countries like Pakistan can be attributed to a lack of adequate surgical facilities and the high cost of surgeries [8]. According to Pakistan Children Heart Foundation, there is not a single specialized children’s heart hospital functioning in Pakistan. There are less than 25 trained pediatric cardiologists and only eight pediatric cardiac surgeons in the entire country. In just one of the hospitals, the number of patients waiting in the queue for surgery has crossed 9,000. Every week, 25-30 children are newly registered for surgery [9].

National Institute of Cardiovascular Diseases (NICVD) in Karachi is a specialized, public-sector cardiology hospital that caters to the cardiovascular needs of patients from the two large provinces of Pakistan: Sindh and Baluchistan. It has a 75-bed pediatric unit and a 14-bed pediatric intensive care unit (PICU) for post-surgical management. The institution is dedicated to free-of-cost management of children with CHD. In a study conducted at NICVD in 2016, 1,100 cases of CHD were identified in two months, which translates into a daily occurrence of 18.6 cases [1]. This study and almost all the other CHD-related studies conducted in Pakistan have focused on the clinical spectrum of CHDs and its various types [1,6,8]. The focus of this study is on the variety and frequency of surgical procedures performed in children with CHD and their outcomes.

## Materials and methods

In our study, all surgical records of children admitted in the PICU of NICVD from October 2018 to March 2019 were included. NICVD in Karachi is a 14-bedded, state-of-the-art PICU, which provides high-quality care to critical post-surgical patients. All services in the NICVD PICU are provided free-of-cost.

Patient age, gender, surgical procedure, and outcomes were recorded. Outcomes were classified under either “discharge” or “mortality". For the purpose of data presentation, surgical procedures were grouped into the following sections: 

(i) Closure repair of septal defects: atrial septal defect (ASD), ventricular septal defect (VSD) and associated pulmonary stenosis (PS), and pulmonary arterial hypertension (PAH) and shunt

(ii) Tetralogy of Fallot (TOF) total correction - with or without right-to-left shunt

(iii) Ligation of patent ductus arteriosus (PDA)

(iv) Cardiac shunts: right-to-left shunt, systemic-to-pulmonary shunt, the Blalock-Taussig (BT) shunt, central shunt for PS, PAH, and/or tricuspid atresia (TA)

(v) Valve repair/replacement: mitral and aortic valve repair/replacement

(vi) Glenn procedure: for TA or hypoplastic left heart syndrome with or without shunt 

(vii) Senning procedure: for transposition of the great arteries with or without other defects

(viii) Total anomalous pulmonary venous return (TAPVR) correction

(ix) Pulmonary artery (PA) banding: for pulmonary over-circulation

(x) Permanent pacemaker placement in complete heart block (CHB PPM)

(xi) Pericardial window: for pericardial effusion.

Data were entered and analyzed using Statistical Package for the Social Sciences (SPSS) for Windows (version 22.0, IBM, Armonk, New York, US). Mean and standard deviation were calculated for continuous variables including age. Frequencies and percentages were calculated for categorical variables including gender, procedure, and outcome.

## Results

Surgical records of 537 patients were extracted for the purpose of our study, which accounted for 89.5 of post-operative patients in the PICU per month and three per day. The mean age of the patients was 6.86 years (range: 8 days - 30 years). There were 230 (42.8%) females with a mean age of 7.4 years (range: 8 days - 30 years). There were 307 (57.2%) males with a mean age of 6.4 years (range: 3 months - 28 years).

Table 1 shows the detailed spectrum of surgical procedures and their outcomes.

**Table 1:** Frequency of surgical procedures and their outcomes in management of CHD. * Including closure/repair of atrial and ventricular septal defects with or without pulmonary stenosis, pulmonary artery banding, and pulmonary artery hypertension. ** Including total correction with or without right-to-left shunt. $ Including right-to-left shunt, systemic-to-pulmonary shunt, Blalock-Taussig shunt, and central shunt. € Including aortic valve, mitral valve, and dual valve replacement. Ᵽ Including coarctation of aorta repair, chest tube passage, subaortic membrane excision, tumor resection, pericardiectomy, infundibulectomy, arterial switch for transposition of the great arteries (TGA) or double switch for ’congenitally corrected’ TGA, and aortic root replacement procedure. Abbreviations: CHB PPM - complete heart block permanent pacemaker; Dis - discharge; F - female; M - male; MT - mortality; PA - pulmonary artery; PDA - patent ductus arteriosus; TAPVR - total anomalous pulmonary venous return; TC - total correction; TOF - Tetralogy of Fallot.

Procedure	Frequency (%)	Gender (%)	Age (years)	Outcome (%)
Closure/repair of septal defects*	188 (35.0%)	M: 98 (52.1%)	M: 6.2	Dis: 181 (96.3%)
		F: 90 (47.8%)	F: 8.3	MT: 7 (3.7%) (5 F; 2 M)
TOF correction**	161 (29.9%)	M: 103 (63.9%)	M: 7.2	Dis: 153 (95.0%)
		F: 58 (36.1%)	F: 8.1	MT: 8 (5.9%) (1 F; 7 M)
PDA ligation	39 (7.3%)	M: 19 (48.7%)	M: 3.3	Dis: 38 (97.4%)
		F: 20 (51.3%)	F: 4.8	MT: 1 (2.6%) (M)
Cardiac shunt^$^	35 (6.5%)	M: 21 (60.0%)	M: 3.1	Dis: 30 (85.7%)
		F: 14 (40.0%)	F: 3.3	MT: 5 (14.3%) (1 F; 4 M)
Valve repair/replacement^€^	31 (5.7%)	M: 16 (51.6%)	M: 14.1	Dis: 28 (90.3%)
		F: 15 (48.4%)	F: 11.8	MT: 3 (9.6%) (1 F; 2 M)
Glenn procedure	16 (2.9%)	M: 10 (62.5%)	M: 6.9	Dis: 15 (93.8%)
		F: 6 (37.5%)	F: 4.7	MT: 1 (6.2%) (M)
Senning procedure	10 (1.8%)	M: 7 (70.0%)	M: 1.4	Dis: 10 (100.0%)
		F: 3 (30.0%)	F: 1.8	MT: 0
TAPVR repair/TC	10 (1.8%)	M: 5 (50.0%)	M: 0.6	Dis: 10 (100.0%)
		F: 5 (50.0%)	F: 1.7	MT: 0
PA band	8 (1.4%)	M: 10 (62.5%)	M: 0.3	Dis: 6 (75.0%)
		F: 6 (37.5%)	F: 1.2	MT: 2 (25.0%) (M)
CHB PPM	6 (1.1%)	M: 2 (33.3%)	M: 12.5	Dis: 6 (100.0%)
		F: 4 (66.7%)	F: 9.3	MT: 0
Pericardia window	6 (1.1%)	M: 3 (50.0%)	M: 3.8	Dis: 6 (100.0%)
		F: 3 (50.0%)	F: 10	MT: 0
Others^Ᵽ^	27 (5.0%)	M: 18 (66.7%)	M: 8.4	Dis: 25 (92.6%)
		F: 9 (33.3%)	F: 5.7	MT: 2 (7.4%) (1 F; 1 M)

Among procedures to repair septal defects, VSD repair was the most commonly performed procedure (n = 107/188; 56.9%), followed by ASD repair (n = 68/188; 36.2%), and atrioventricular septal defect (AVSD) repair (n = 13/188; 6.9%). VSD was the second most commonly treated lesion overall (n = 107/537; 19.9%) after TOF (29.9%). Among the cases of TOF, 68 (42.2%) were managed with total correction and in the remaining 93 (57.7%), shunts were placed.

The overall mortality rate was 5.4% (n = 29). The mean age of the patients was 4.9 years (range: 0.25 - 20 years). There were 20 (68.9%) male children and nine (31.1%) female children among the mortalities. There were eight (27.5%) TOF-related mortalities: five (62.5%) cases of TOF TC, one (12.5%) case of TOF with pulmonary atresia, and two (25%) cases of TOF correction with central shunt placement. Three (10.3%) deaths were related to post-transcatheter ASD closure, one (3.4%) to VSD closure, one (3.4%) to VSD closure with irreversible PAH, and one (3.4%) to VSD plus PS repair with right-to-left shunt. Two (6.9%) deaths were related to dextro-TGA and both of them were complicated by VSD. And one (3.4%) death was post-VSD plus pulmonary atresia repair and one (3.4%) was post-mitral valve repair. There were three (10.3%) deaths post right-to-left shunt placement, one (3.4%) death after BT shunt, one (3.4%) after central shunt, and one (3.4%) after Glenns shunt placement. Four (13.8%) deaths were related to PA banding and PDA ligation, and one (3.4%) was related to arterial switch.

## Discussion

CHD remains the most common congenital birth defect globally. It has a higher mortality rate when compared to other birth anomalies. Concrete data is absent regarding its incidence and prevalence. However, most recent statistics have recorded an increasing trend in CHD occurrence. Its prevalence has escalated from 4.5 per 1,000 live births in 1970-74 to 9.4 per 1,000 live births in 2010-17 (P <0.001). The prevalence of mild lesions including ASD, VSD, and PDA showed an increasing trend throughout the study period, indicating improved and early diagnosis. This meta-analysis also reported the highest average prevalence of mild CHD lesions from Asia compared to other parts of the world (9.3 per 1,000 live births) [2,3].

This six-month analysis from NICVD (October 2018 - March 2019) reported 537 pediatric cardiac surgeries, which approximates to 1,074 surgeries per year (2018-19). Another study conducted at NICVD from October 2013 to September 2015 showed that 1,004 pediatric cardiac surgeries were performed at this institution [10]. This reveals that over a period of three years (2015-18), this hospital has doubled its capacity and is performing twice the number of surgeries. It also translates into increased incidence, presentation, and severity of CHD in this region. The limitation of this study is its retrospective method of data collection. It may also reflect a bias relating to human error in coding. Furthermore, due to the lack of time and funds, data pertaining to only six months could be analyzed. 

Among other studies from Pakistan, Masood et al. have reported a 33% prevalence of VSD and 17% of TOF [11]. Similarly, Mohammad et al. have reported a 29.9% prevalence of VSD, 25.4% of ASD, and 11.2% of TOF [12]. Pathan et al., from NICVD in 2016, reported TOF and VSD to be the most common surgically managed pediatric cardiac lesions. However, our study shows that the rate of TOF correction has come down from 42.3% to 29.9%. The rate VSD remained steady (20.9% vs. 19.9%) nonetheless. Even with double the number of pediatric cardiac surgeries in the course of three years, there has been an overall 2.5% decline in the mortality rate (8% vs. 5.5%) [10]. In another study from a private tertiary care institute in Pakistan, the mortality rate reported in pediatric cardiac surgeries was 6-8.7% [13]. Studies from other low- and middle-income countries have also reported higher mortality among children after cardiac surgery: Indonesia - 12.8% [14]; Iran - 12.4% [15]; Guatemala - 10.7% [16]; and India - 11.5% [17]. Contrary to this, high-income countries have reported less than 5% mortality after pediatric cardiac surgery [18]. 

In a low-to-middle income and low-resource country like Pakistan, we have managed to reduce the mortality rate related to CHDs, thanks to advancements in pediatric cardiac critical care (PCCC) services. In a nationwide survey published in 2014, there were 16 PICUs reported to be functioning across Pakistan, out of which three are purely cardiac PICUs. According to this survey, one PICU bed is available for every 500,000 Pakistani children under the age of 14. The statistics from developed countries tell a far different story (one bed/10,000 children in the United States and one bed/1,000 children in the United Kingdom) [19]. The importance of PCCC services have been repeatedly highlighted in the literature [20]; particularly for Asian countries like India and Pakistan, where the incidence of CHD has already been reported to be higher than in other parts of the world [2,3]. The institution of PCCC and induction of trained staff in these setups have played a crucial role in reducing the overall mortality over a period of three years despite an increase in the total number of surgeries performed [10]. High-quality cardiac ICUs provide efficient post-surgical services to children with CHD and enable the critical-care specialists to address high morbidity and mortality after critical cardiac surgeries. One of the key concerns with pediatric cardiac surgery in low-to-middle income countries is the competing priorities, which is multifactorial and may be due to lack of financial resources or trained human resources. In such a scenario, PCCC units play a significant role in reducing the massive rate of preventable mortalities [20].

In order to address this burden, PCCC training and specialization has to be prioritized in large cardiac care centers. Focused training of pediatric cardiac nurses, cardiologists, and critical-care specialists, anesthesiologists, and cardiothoracic surgeons must be encouraged.

## Conclusions

With the rising incidence of CHD in Asian countries such as Pakistan, establishment of pediatric cardiac critical care units have allowed for the provision of quality post-surgical care. Correction of TOF and closure of VSD were the most commonly performed surgical procedures. Despite limited resources, the mortality rate was reported to be only 5%. Adequate financial resources, advance medical equipment, and a team-oriented approach by well-trained pediatric cardiac critical-care nurses and pediatric cardiologists have ensured a favorable outcome in the treatment of most of the children suffering from CHDs in Pakistan.
